# Relationship between corporate culture, service quality, and customer satisfaction of food and beverage franchise enterprises

**DOI:** 10.12688/f1000research.154431.1

**Published:** 2025-03-05

**Authors:** Duyen Chau Thi Le, Liem Nguyen Thanh, Linh Huynh Giao

**Affiliations:** 1 Business Admistration, Can Tho University, Can Tho, +84, Vietnam; 2 Business Admistration, Tra Vinh University, Trà Vinh, +84, Vietnam

**Keywords:** Corporate culture, Organizational culture, Service quality, Franchise enterprises, Food and Beverage.

## Abstract

**Background:**

This study examines the intricate relationship between corporate culture, service quality, and customer satisfaction in franchise businesses operating in the Mekong Delta region of Vietnam. Given the increasing significance of corporate culture in shaping business performance, this research aims to uncover its direct and indirect effects on customer satisfaction.

**Methods:**

The study utilizes a dataset of 207 observations and employs a rigorous analytical framework. The methodology includes descriptive statistics, Cronbach’s Alpha for reliability assessment, Exploratory Factor Analysis (EFA) for dimensionality reduction, Confirmatory Factor Analysis (CFA) for model validation, and Structural Equation Modelling (SEM) for hypothesis testing.

**Results:**

The findings highlight the significant influence of corporate culture on service quality, which subsequently impacts customer satisfaction. Moreover, service quality is identified as a mediating factor, demonstrating an indirect pathway through which corporate culture enhances customer satisfaction. These results underscore the interconnectedness between organizational culture and service performance, particularly in franchise settings within emerging economies.

**Conclusions:**

This study offers practical insights for franchise business operators seeking to enhance their competitive advantage. Establishing a strong corporate culture that emphasizes customer orientation, innovation, and employee engagement is crucial. Additionally, investment in service quality—through employee training, process improvements, and customer-centric policies—is essential for fostering customer satisfaction and loyalty. This research contributes to the literature on organizational behavior and service management by shedding light on the mechanisms through which corporate culture influences customer satisfaction via service quality. It provides a nuanced understanding of these dynamics in the underexplored context of franchise businesses in Vietnam, offering valuable implications for both academia and practice.

## Background

Corporate culture is widely recognized as a fundamental component in shaping the sustainability and competitive advantage of enterprises ( Alamri et al., 2021; Al-Dmour et al., 2019; Amankwah-Amoah & Adomako, 2019). It represents the core values that define an organization’s identity, influencing both internal operations and external perceptions. A strong corporate culture fosters brand differentiation and plays a pivotal role in long-term success ( Alalwan et al., 2020; Kabir et al., 2020; Kotler & Gertner, 2011). In the franchise business model, corporate culture is particularly critical, as it ensures consistency across different locations and strengthens customer trust ( Nguyen Quang Thu & Nguyen Dai Phuoc Tien, 2010).

Vietnam has emerged as a key market for franchising, attracting numerous international brands such as McDonald’s, Baskin Robbins, Pizza Hut, Burger King, Lotteria, and BBQ Chicken. The franchising sector in Vietnam is diverse, with fast food chains and restaurants accounting for 41.31% of total franchise operations, followed by retail stores (15.49%), fashion (14.08%), and education and training (11.47%). Vietnam’s dynamic consumer market, driven by a population of over 99 million, high consumption rates, and increasing incomes, has made it an attractive destination for foreign investors ( Ministry of Industry and Trade, 2022).

Given this context, this study investigates the relationship between corporate culture, service quality, and customer satisfaction within the franchising sector in Vietnam. Specifically, it aims to analyze the impact of corporate culture on service quality and customer satisfaction and provide managerial implications for enhancing corporate culture and competitive advantage in the food and beverage (F&B) franchise industry.

### Theoretical background and research model

According to Schein (1992), corporate culture comprises three levels: artifacts, espoused values, and underlying assumptions. The first level, artifacts, includes visible and tangible elements that describe the physical environment and social activities within an organization, such as distinctive architecture and corporate appearance; celebrations, rituals, cultural activities; slogans and language; symbols, traditional songs, and uniforms. The second level, espoused values, encompasses vision, mission, core values, objectives, and strategies. The third level, underlying assumptions, consists of deeply held beliefs, perceptions, thoughts, and feelings acknowledged within the organization. These assumptions are challenging to change and significantly influence work style, communication decisions, and behavior.

Upon reviewing various models used to study corporate culture, it is observed that Edgar H. Schein’s model aligns well with the research direction, and Duong Thi Lieu has also employed this model in her studies. The model illustrates the perception of cultural values within an organization, reflecting the tangible and intangible, visual and non-visual expressions of these values. Schein (2004) defines corporate culture as the set of values, norms, and fundamental beliefs accumulated during the organization’s interactions with the external environment and internal integration. These values and norms are established over time and are conveyed to new members as the correct way to approach, think, and resolve issues encountered.

Additionally, Dean (2005) posits that corporate culture is simply how an organization structures and conducts its activities. Another perspective by Hoai (2009) views corporate culture as the values and philosophies agreed upon by all members of the organization, significantly influencing its business operations and creating a unique corporate identity. Corporate culture is a system of shared meanings, values, dominant beliefs, perceptions, and thinking methods widely influencing members’ actions ( Schein, 2004; Quan, 2011). It facilitates members in recognizing the distinctiveness that the enterprise aspires to achieve. Overall, definitions of corporate culture generally consider it as the entirety of cultural values built throughout an enterprise’s existence and development, governing the emotions, thoughts, and behaviors of all its members. This creates differentiation between enterprises and is regarded as the unique tradition of each organization.

Service Quality: Lewis and Mitchell (1990) define service quality as the extent to which the service delivered meets or exceeds customer expectations. Cronin and Taylor (1992) describe service quality as the consumer’s perception of the service. Service quality serves as a precursor to customer satisfaction. Zeithaml (1988) defines service quality as the consumer’s overall assessment of the excellence and superiority of a service. It reflects an attitude resulting from a comparison between expected and perceived service outcomes. Ekinci (2003) argues that enhancing service quality will lead to increased customer satisfaction. Nguyễn Thị Mai Trang and Trần Xuân Thu Hương (2010) assert that quality must be evaluated from the perspective of service users or consumers. Service quality arises from consumers’ perceptions of service outcomes.

The seminal work of Parasuraman et al. (1988) significantly contributed to the understanding of service quality. They define service quality as the discrepancy between consumer expectations of a service and their perceptions of the service received. This team pioneered the use of both qualitative and quantitative research to develop and validate a measurement scale for service quality, known as the SERVQUAL scale. The SERVQUAL scale, which was refined and validated across various service sectors, ultimately included 22 items measuring five dimensions of service quality: reliability, responsiveness, assurance, tangibles, and empathy.

The SERVQUAL model measures service quality based on the gap between customer expectations and perceptions. It is recognized as a valuable theoretical and practical measurement tool, extensively tested and utilized across different fields. To address limitations in SERVQUAL, Cronin and Taylor (1992) introduced the SERVPERF (Service Performance) model, which eliminates the expectation component and focuses solely on customer perceptions with five service quality factors: reliability, responsiveness, service competence, empathy, and tangibles, using 22 observed variables. The SERVPERF model is considered a convenient and clear method for measuring service quality, as it reduces the number of questions and thus shortens research time while being well-received by respondents ( Brady et al., 2002). However, it does not capture the attributes of service that customers may have high expectations for.

Customer Satisfaction: Customer satisfaction has been defined in various ways. According to Parasuraman et al. (1988), customer satisfaction is the response to the perceived discrepancy between known experiences and expectations. Kotler and Armstrong (2001) describe satisfaction as the degree of a person’s state or feeling resulting from comparing the outcomes of consuming a product with their expectations. Expectations, in this context, are seen as human desires or anticipations, arising from personal needs, prior experiences, and external information such as advertisements and word-of-mouth from friends and family. Zeithaml et al. (2006) define satisfaction as the customer’s evaluation of a product or service in terms of whether it meets their needs and expectations. Thus, customer satisfaction is derived from experiences, particularly those accumulated from purchasing and using products or services. Oliver et al. (1985) posits that satisfaction is a consumer’s response to having their desires fulfilled. This definition implies that satisfaction reflects the degree to which a product or service meets consumer desires, encompassing both exceedance and shortfall of expectations.

Factors Influencing Customer Satisfaction: (1) Service Quality: Service quality is defined as the extent to which a service meets customer needs and expectations ( Lewis & Mitchell, 1990). Edvardsson, Thomsson, and Ovretveit (1994) describe it as the extent to which a service meets customer expectations and fulfills their needs. (2) Service Price: This depends on various factors such as service quality, brand reputation, and additional services like payment discounts and warranties. In contemporary consumer trends, customers are often willing to pay higher prices for services that meet their needs effectively. Therefore, price also has a significant impact on satisfaction. (3) Brand: The concept of brand is intrinsically linked to the intangible values in consumers’ minds. It is a crucial factor directly affecting customer satisfaction and is positively related to satisfaction with a product or brand. In the mobile information sector, for instance, brand plays a critical role in customers’ choice of network and service options. (4) Promotional Advertising: In a competitive market, promotional programs create differentiation in customer care and appreciation, significantly impacting customer satisfaction. (5) Value-added Services: These are factors that distinguish services among market providers. Companies offering more distinctive value-added services tend to attract more customer interest and achieve higher satisfaction levels. (6) Convenience: Convenience affects both access to and usage of services. Companies that provide easier access to their services or offer more memorable names, simpler usage procedures, and diverse and reasonable payment methods generally achieve higher customer satisfaction. (7) Customer Support: When service quality is similar across providers, customer support becomes a competitive advantage. It includes customer support systems and processes for handling complaints and disputes. Effective customer support builds customer trust, reassures them about using the service, and enhances overall satisfaction.

**Figure 1.**
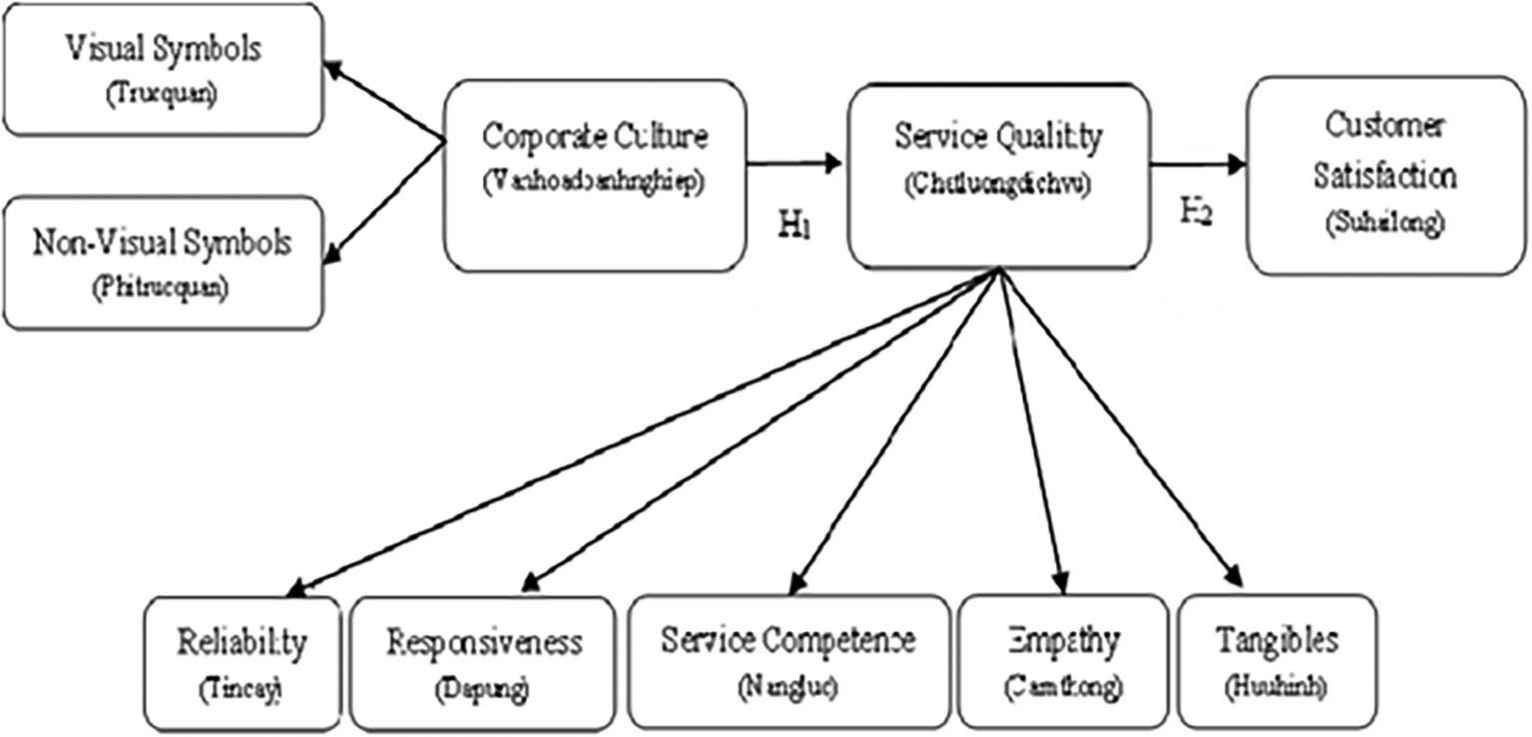
Research model. Source: Analysis results from customer survey data, 2022.

From the review of previous studies, this research has adjusted and supplemented to establish the following appropriate research model ( Figure 1)

Hypotheses are proposed as follows:

H _1_ *Corporate Culture style has a positive impact on Service Qualitity*

H _2_ *Service Qualitity has a positive impact on Customer Satisfaction*

In summary, the factors of corporate culture include both tangible and intangible elements; for service quality, the factors include reliability, responsiveness, service competence, empathy, and tangibles; and customer satisfaction.

## Methods

### Data collection and sampling

Primary data were collected through surveys administered to customers who had previously used products or services at establishments such as HighLands Coffee, King BBQ Buffet, and Gong Cha in Can Tho, Viet Nam. A structured questionnaire was used for this purpose. According to Habing (2003), the minimum sample size for observations should be 4-5 times the number of independent variables. Additionally, Hair et al. (1998) recommend that the minimum sample size should be 50, with an ideal size of 100, and an observation-to-variable ratio of 5:1. This implies that for each measurement variable, five observations are required. In this study, there are 33 measurement variables, so the minimum required sample size is calculated as 33 x 5 = 165 observations. Furthermore, as exploratory factor analysis (EFA) is employed, Gorsuch (1983) suggests a minimum sample size of 200 observations. To ensure sufficient and valid data while minimizing errors during the survey process, the author proposed a sample size of 212 observations.

Due to time constraints and to facilitate the sampling process, the study utilized a non-probability convenience sampling method, enabling easier access to respondents. The questionnaire employed a 5-point Likert scale to measure respondents’ perceptions of the impact of various factors, where 1 represented “strongly disagree,” 2 “disagree,” 3 “neutral,” 4 “agree,” and 5 “strongly agree.”

The questionnaire comprised three main sections:

1. **Screening questions**: This section included two closed-ended questions to filter respondents who matched the research objectives.
2. **Demographic information**: This section consisted of seven questions designed to collect personal information about the respondents to support the research process.
3. **Core content**: This section contained 39 questions related to factors such as corporate culture, service quality, and customer satisfaction.

**Ethical considerations**. The study used a convenience sampling method for ease of respondent accessvalidity and minimizeThus, while the minimum sample size for this study is 165 observations, a larger sample size of over 200 observations was selected torecommendsusedAdditionally, given that required in this study, With in the study, 5every, meaningideally 100, ofsuggest a employed to gather necessary informat.

In this study, informed consent was obtained from all participants in accordance with the ethical standards for research involving human subjects. Participants were assured of the voluntary nature of their involvement and their right to withdraw from the study at any time without penalty. To maintain confidentiality, all personal data were anonymized. This research was reviewed and approved by the **Board of Directors of the School of Economics, Can Tho University, Vietnam**, on February 4, 2022. While an official Ethics Committee has not yet been established at the institution during the time of this study, the approval from the Board of Directors ensured adherence to ethical principles. All procedures performed in this study were conducted in compliance with the ethical principles outlined in the **Declaration of Helsinki**. Standardized protocols for data collection and analysis were adopted to ensure objectivity and minimize potential biases in the interpretation of the results. Adherence to these guidelines ensured rigor in the development and presentation of the research, promoting transparency and replicability.

### Measurement

The study employed descriptive statistics to summarize and present the data, providing a comprehensive overview of the study population. Frequency analysis was conducted using frequency distribution tables, which summarize data by categorizing it into various factors based on the frequency of occurrences in the database. This method allows for comparisons of proportions and highlights the data’s key features.

Subsequently, the study applied Structural Equation Modeling (SEM) to evaluate the overall model fit, using indicators similar to those employed in Confirmatory Factor Analysis (CFA). A model is deemed a good fit if TLI and CFI are ≥ 0.9, CMIN/df ≤ 2, and RMSEA ≤ 0.08 ( Bentler & Bonett, 1980; Bullock et al., 1994). SEM provides several advantages over traditional multivariate regression methods for hypothesis testing and model evaluation, as it accounts for measurement errors, integrates latent constructs with their measurements, and simultaneously examines independent or combined measures within a theoretical framework.

**Bootstrap Testing in SEM:** Bootstrap analysis was conducted to validate model estimates by comparing bootstrap and original sample estimates. A critical ratio (C.R.) > 1.96 with a p-value < 0.05 indicates statistical significance at the 95% confidence level. Lower values suggest non-significance. SEM analysis specifies the relationships between latent variables and is advantageous because it accommodates measurement errors and integrates latent constructs with observed data. In quantitative research methods using sample-based approaches, the sample is typically divided into two subsamples—one for estimating model parameters and the other for reevaluation. Alternatively, the study could be repeated with a different sample. However, these approaches are often impractical due to the large sample size required for SEM, leading to significant time and cost constraints ( Anderson & Gerbing, 1988). In such cases, the bootstrap method is a suitable alternative ( Schumacker & Lomax, 1996). Bootstrap is a resampling method with replacement, where the initial sample serves as the population. The bootstrap procedure involves generating N resamples, calculating estimates from each, and averaging the results. This average tends to approximate the population estimates. A smaller discrepancy between bootstrap-estimated values and the original model estimates indicates the reliability of the model estimates.

## Result

The target population for this study consists of customers who have used products from establishments such as HighLands Coffee, King BBQ, and Gong Cha. To achieve the required sample size, a total of 212 customers were surveyed. After excluding unreliable responses, the final sample size for the analysis was 207. Specifically, the details of the sample are as follows:

The survey results reveal that the majority of respondents are female (65.22%), surpassing the male proportion. This preference may be attributed to women’s tendencies to enjoy coffee and tea at HighLands Coffee for its robust flavors, which help them stay alert during long workdays, or their fondness for the sweet and creamy offerings of Gong Cha. In contrast, men are more likely to visit these establishments for professional meetings or social gatherings, valuing the establishments’ refined and modern atmospheres, or they may be coffee enthusiasts. Additionally, most patrons of King BBQ visit with family or friends on weekends or special occasions. This explains why King BBQ’s promotional strategies often target customer volume rather than product discounts ( Table 1).

**Table 1.** General information of respondents.

Criteria		Frequency (Observations)	Proportion (%)
Gender	Male	72	34.78
	Female	135	65.22
**Total**		**207**	**100**
Number of Times Used (times/month)	1	91	43.96
	2	51	24.64
	>2	65	31.40
**Total**		**207**	**100**

Source: Analysis results from customer survey data, 2022.

Regarding product usage frequency, 31.40% of customers use the products more than twice a month, 24.64% use them twice a month, and the remaining 43.96% use them once a month. This indicates a relatively moderate level of product consumption, possibly due to higher pricing which may not be accessible to all customer segments. Despite efforts to make products more affordable, they have not achieved the broad reach of traditional coffee shops or eateries ( Table 2).

**Table 2.** Exploratory factor analysis results for corporate culture scale.

Variables	Factor	
	1	2
TQ1	0.782	
TQ3	0.740	
TQ4	0.724	
TQ5	0.706	
TQ2	0.703	
PTQ1		0.793
PTQ4		0.770
PTQ5		0.733
PTQ3		0.719
PTQ2		0.621
Eigenvalue		2.060
Total Variance Explained		62.828
Pvalue (Bartlett Test)		0.000
KMO		0.860

Source: Analysis results from customer survey data, 2022.

Service Quality Scale: After testing the reliability of the service quality scale using Cronbach’s Alpha coefficient, 5 factors with 22 observed variables that met the requirements were retained for exploratory factor analysis (EFA). The EFA results for the scale revealed that 5 factors were extracted. The Eigenvalues for the factors were all greater than 1, with the fifth factor having the lowest Eigenvalue of 1.360. The total variance explained was 65.927%, which is greater than 50%, indicating that the EFA is satisfactory and these 5 factors explain 65.927% of the variance in the data. The KMO index was 0.889 (0.5 < 0.889 < 1), suggesting that the data is suitable for confirmatory factor analysis (CFA). Bartlett’s test result was 2,119.133 with a significance level of Sig. = 0.000 < 0.05, indicating that the variables are correlated with each other and satisfy the conditions for factor analysis ( Table 3).

**Table 3.** Exploratory factor analysis results for service quality scale.

Variables	Factor				
	1	2	3	4	5
NL1	0.752				
NL4	0.721				
NL2	0.711				
NL5	0.652				
NL3	0.614				
CT5		0.764			
CT1		0.750			
CT2		0.729			
CT3		0.704			
CT4		0.615			
TC2			0.836		
TC3			0.751		
TC1			0.747		
TC4			0.730		
HH3				0.832	
HH2				0.798	
HH4				0.712	
HH1				0.647	
DU3					0.772
DU4					0.762
DU1					0.697
DU2					0.576
Eigenvalue					1.360
Total Variance Explained					65.927
Pvalue (Bartlett Test)					0.000
KMO					0.889

Source: Analysis results from customer survey data, 2022.

Customer Satisfaction Scale: After testing the reliability of the customer satisfaction scale using Cronbach’s Alpha coefficient, 1 factor with 5 observed variables met the requirements. The EFA results for the customer satisfaction scale are presented in Table 4.

**Table 4.** Exploratory factor analysis results for customer satisfaction scale.

Variables	Factor
	1
HL3	0.797
HL4	0.792
HL2	0.782
HL5	0.781
HL1	0.759
Eigenvalue	3.448
Total Variance Explained	68.962
Pvalue (Bartlett Test)	0.000
KMO	0.887

Source: Analysis results from customer survey data, 2022.

The EFA results for the customer satisfaction scale indicate that one factor was extracted. The Eigenvalues for this factor is 3.448, which is greater than 1. The total variance explained is 68.962%, which is higher than the 50% threshold, indicating that the EFA analysis meets the required standards and that this factor explains 68.962% of the data variance. The KMO measure is 0.887 (0.5 < 0.887 < 1), suggesting that the data is suitable for conducting Confirmatory Factor Analysis (CFA). Bartlett’s test result is 520.809 with a significance level of Sig. = 0.000 < 0.05, confirming that the variables are correlated and meet the conditions for factor analysis.

The study employs several fit indices to assess model adequacy, including Chi-square, Chi-square divided by degrees of freedom (CMIN/df ), Comparative Fit Index (CFI), Tucker-Lewis Index (TLI), and Root Mean Square Error of Approximation (RMSEA). A model is considered suitable if the Chi-square test yields a p-value < 0.05, CFI and TLI values range from 0.9 to 1, CMIN/df is less than 2, and RMSEA is below 0.08. These criteria indicate that the model fits the market data adequately.

**Figure 2.**
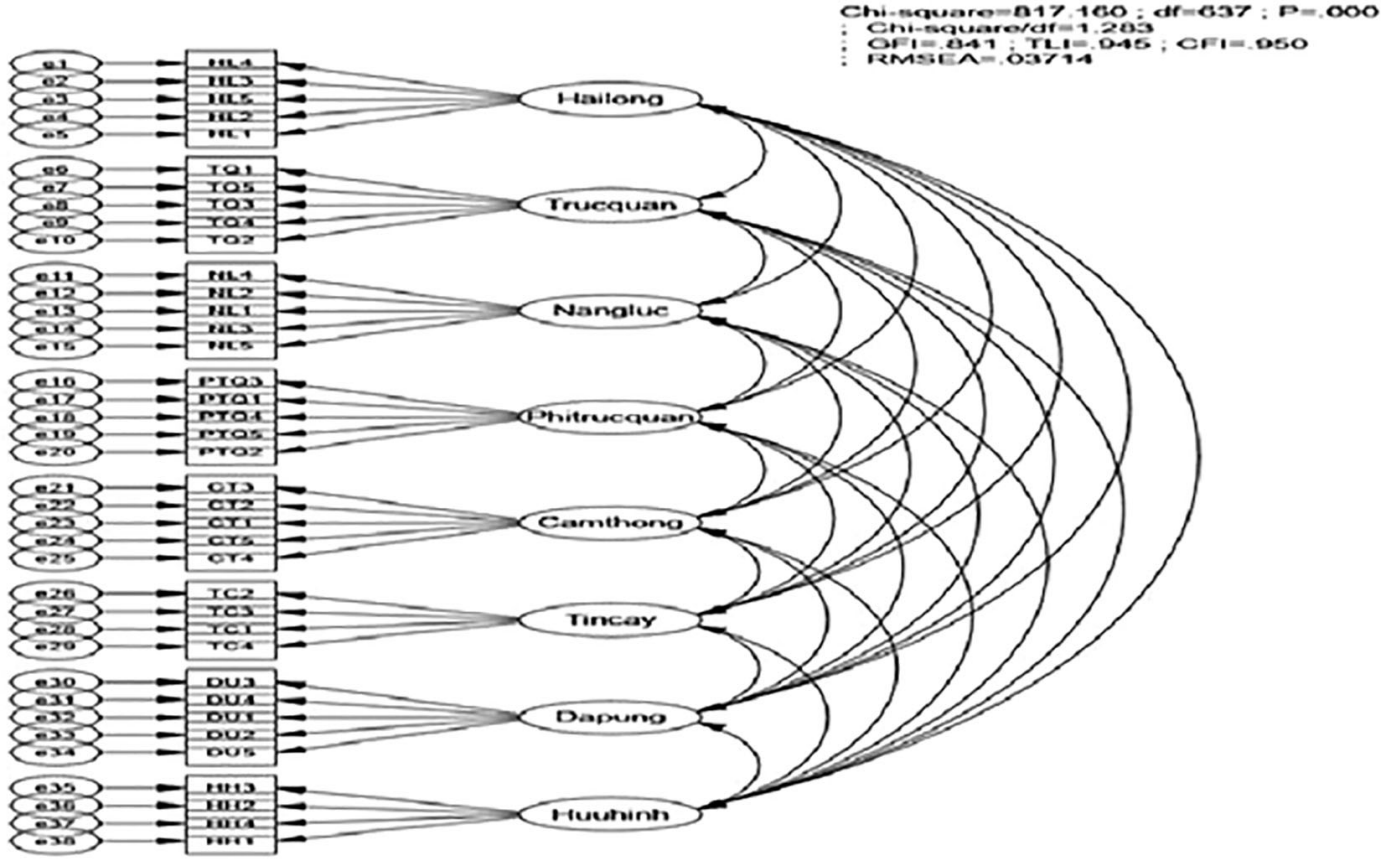
CFA results for model constructs (Standardized). Source: Analysis results from customer survey data, 2022.

The Confirmatory Factor Analysis (CFA) results indicate the following: The Chi-square statistic is 767.196 with 601 degrees of freedom, and the P-value is 0.000 (< 0.05). Additionally, other fit indices demonstrate that the model fits the market data well: GFI = 0.846 (acceptable as it exceeds 0.8), TLI = 0.949, CFI = 0.954 (both > 0.9), and RMSE =0.037 (< 0.08). Therefore, it can be concluded that the model achieves a satisfactory fit with the market data ( Figure 2).

Unidimensionality: The model’s fit with the data indicates that the scale achieves unidimensionality. According to Steenkamp and Van Trijp (1991), the model’s fit with the market data provides necessary and sufficient conditions for the set of observed variables to exhibit unidimensionality, except in cases where error terms between observed variables are correlated. Given the relative fit indices and the absence of error correlations among observed variables within each scale, it can be concluded that the model demonstrates unidimensionality.

Convergent Validity: The model achieves convergent validity when standardized loadings are 0.6 or higher ( Anderson & Gerbing, 1988) and are statistically significant with p-values < 0.05. Table 2 shows that all loadings meet the standard (> 0.6) and are statistically significant with p-values = 0.000. Therefore, it can be concluded that the scale variables exhibit convergent validity.

Reliability Assessment: The Cronbach’s Alpha coefficients for the factors all exceed the required threshold (> 0.6). The variance extracted for the factors is greater than 50%, and the composite reliability for the factors also meets the standard, being greater than 0.6. Thus, the scale meets the reliability standards.

**Figure 3.**
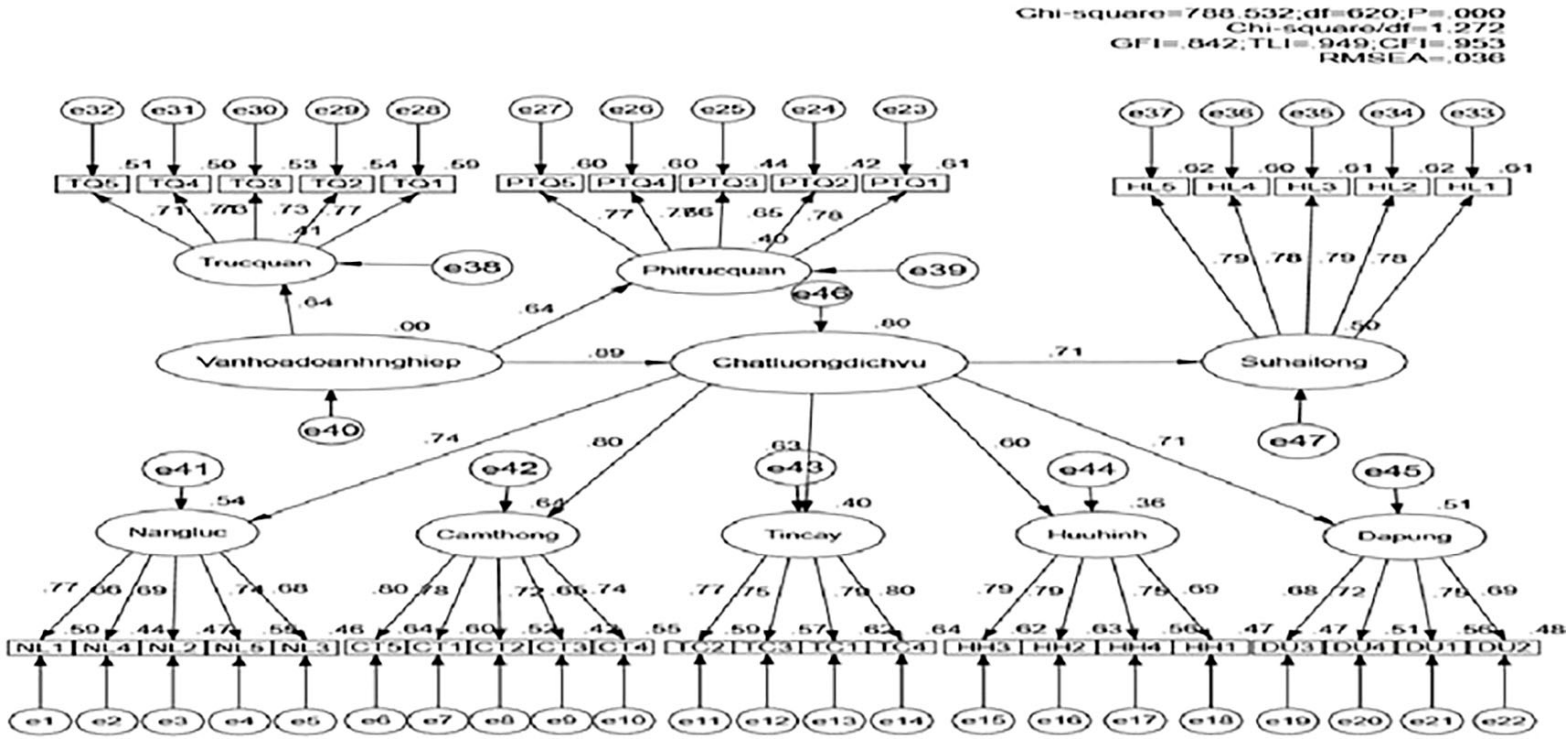
Results of the SEM analysis for the theoretical model (Standardized). Source: Analysis results from customer survey data, 2022.

Testing the Theoretical Model with SEM: The theoretical model was evaluated using Structural Equation Modeling (SEM). The model yielded a Chi-square value of 788.532 with 620 degrees of freedom (p = 0.000). The Chi-square/df ratio of 1.272, which is less than 2, indicates an acceptable fit to the market data. Additionally, other fit indices also meet the required standards: GFI = 0.842 (exceeding the acceptable threshold of 0.8); TLI = 0.949; CFI = 0.953; and RMSEA = 0.036 (below the threshold of 0.08). These results confirm that the model is well-suited to the market data ( Figure 3).

The parameter estimates (standardized) indicate that the standardized weights for the key factors are positive, demonstrating that organizational culture has a positive impact on service quality, with an influence coefficient of 0.892. Additionally, service quality positively affects customer satisfaction, with an influence coefficient of 0.707. Both relationships are statistically significant (p < 0.05) ( Table 5).

**Table 5.** Reliability assessment of measurement scales.

Concept	Component	Numbers	Cronbach’s Alpha	Variance Extracted
Corporate Culture (vanhoadoanhnghiep)	Visual Symbols (Trucquan)	5	0.851	0.535
	Non-Visual Symbols (Phitrucquan)	5	0.849	0.532
Service Quality (chatluongdichvu)	Reliability (Tincay)	4	0.859	0.604
	Responsiveness (Dapung)	4	0.788	0.506
	Service Capability (nangluc)	5	0.833	0.503
	Empathy (Camthong)	5	0.857	0.548
	Tangibles (huuhinh)	4	0.839	0.569
Customer Satisfaction (Suhailong)		5	0.887	0.612

Source: Analysis results from customer survey data, 2022.

Bootstrap methodology will be employed to assess the reliability of estimates in the research model and the quality of regression coefficients within the model. The critical ratio (C.R) values will be compared against the threshold of 1.96, corresponding to the 97.5th percentile of the standard normal distribution (2.5% in one tail, or 5% in both tails combined). If the p-value is less than 5%, it is concluded that the hypothesis of bias being different from zero is statistically significant. Specifically, if C. R > 1.96, this implies a p-value < 5%, indicating that the deviation from zero is statistically significant at a 95% confidence level. Typically, such results are not anticipated in SEM analysis, and conversely, are indicative of expected outcomes ( Table 6).

**Table 6.** Bootstrap estimation results with N=1000.

Relationships	Standardized Estimation	Bootstrap Estimation					
		SE	SE-SE	Mean	Bias	SE-Bias	C.R
Service Quality (chatluongdichvu) <--- Corporate Culture (vanhoadoanhnghiep)	0.892	0.120	0.003	0.898	0.006	0.004	1.500
Customer Satisfaction (Suhailong) <--- Service Quality (chatluongdichvu)	0.707	0.057	0.001	0.709	0.002	0.002	1.000

Source: Analysis results from customer survey data, 2022.

The results of the Bootstrap test indicate that the absolute values of the Critical Ratios (CR) are all less than 1.96. Therefore, the deviations from zero are not statistically significant at the 95% confidence level, suggesting that the estimates in the model are reliable.

The results of the analysis of the relationships between the hypotheses are presented in Table 7. The findings indicate that hypotheses H _1_ and H _2_, concerning the relationships between the concepts within the research model, are supported with statistical significance (p < 0.005).

**Table 7.** Results of hypothesis testing for conceptual relationships.

Hypothesis	Relationships	Standardized Estimation	P	Conclusion
H _1_	Service Quality (chatluongdichvu)<--- Corporate Culture (vanhoadoanhnghiep)	0.892	0.000	Confirmed
H _2_	Customer Satisfaction (Suhailong)<--- Service Quality (chatluongdichvu)	0.707	0.000	Confirmed

Source: Analysis results from customer survey data, 2022.

The acceptance of Hypothesis H _1_, which posits that “There is a relationship between corporate culture and service quality in franchise businesses in Can Tho,” is confirmed. The results indicate a positive moderate relationship between corporate culture and service quality, with an estimated coefficient of 0.892. This suggests that a well-established corporate culture enhances the service quality of businesses, thereby increasing customers’ perceived value of the service. One of the most prominent cultural symbols that customers recognize is the brand logo and employee uniforms, which are highly appreciated by customers. When employees wear company uniforms, it creates a pleasing and comfortable impression for customers, fostering a positive connection with the business. Proper attire also helps employees understand their roles and perform more effectively, benefiting the company. These simple symbols illustrate the positive relationship between corporate culture and service quality. Improving service quality can be achieved in various ways, and leveraging corporate culture is an effective approach that can reduce management costs and enhance business performance. Building a strong corporate culture can attract new customers and retain loyal ones.

The acceptance of Hypothesis H _2_, which posits that “There is a relationship between service quality and customer satisfaction in franchise businesses in Can Tho,” is confirmed with an estimated coefficient of 0.707, indicating a positive relationship between service quality and customer satisfaction. This implies that improvements in service quality positively impact customer satisfaction. However, the positive relationship, though significant, is not very high, suggesting that businesses may not fully meet customer needs, which diminishes their satisfaction. This finding highlights existing gaps in service quality provision and the need for businesses to adopt measures to enhance service quality, with corporate culture being a relatively effective solution to consider.

The research data analysis indicates that corporate culture affects service quality, and service quality, in turn, influences customer satisfaction in franchise businesses. The study confirms that the measures for corporate culture, service quality, and customer satisfaction are validated and meet the required standards. The impact of corporate culture on service quality and customer satisfaction was examined and validated through the SEM model. Additionally, the theoretical model was tested using the Bootstrap method to ensure the reliability of the estimates. The study reveals that corporate culture directly impacts service quality, which, in turn, affects customer satisfaction. This demonstrates an indirect relationship where corporate culture influences customer satisfaction through service quality.

## Conclusion

The research results clearly demonstrate that when corporate culture is prioritized and well-developed, service quality improves, leading to higher customer satisfaction. Therefore, enhancing corporate culture contributes to improving service quality and customer satisfaction. This necessitates that franchise businesses recognize the role and importance of continuously perfecting and further developing their unique corporate culture in the future. Developing corporate culture is a crucial strategy for enhancing the competitive capacity of businesses. It requires tools for reviewing and comprehensively assessing corporate culture, policies, and management philosophies, especially when businesses face volatile environments or economic crises. By studying the relationship between corporate culture, service quality, and customer satisfaction, it is evident that there are statistically significant impacts among these concepts. Both theoretical and practical research demonstrates that corporate culture is an intangible asset, a spiritual resource, and a driving force for sustainable business development. Corporate culture is not only a method and tool for management but also a valuable asset of the business, necessitating scientific leadership and management. It requires tasks of building, organizing, implementing, and integrating into business activities and life, as well as applying in strategic and human resource management for sustainable development. Establishing and managing a strong, enduring corporate culture is a fundamental condition for sustainable business development and contributing value to society.

Measuring service quality provides qualitative and quantitative benefits to businesses. Achieving high service quality levels enhances customer loyalty, increases market share, returns on investment, reduces costs, and ensures competitive advantages. The research results indicate a close and strong relationship between service quality and customer satisfaction. Service quality relates to service delivery, while customer satisfaction is the fulfillment assessed after using the service. Factors influencing service quality also impact customer satisfaction. Thus, service quality is considered a precursor that determines customer satisfaction. The theoretical basis for identifying the elements of the concepts is still loosely defined, as it mainly adapts predefined concepts and elements to fit the research context of franchise businesses. The research results are limited in that they do not comprehensively reflect the factors constituting culture, service quality, and customer satisfaction on a larger scale. The analysis results have many limitations when generalized to the whole population.

### Ethical consideration

This study was conducted in adherence to the ethical principles outlined in the Declaration of Helsinki. The research received approval from the **Board of Directors of the School of Economics, Can Tho University, Vietnam**, on February 4, 2022. As the institution has not yet established an official ethics committee at the time of the study, no formal approval number was issued.

Informed consent was obtained from all participants before their involvement in the study. Participants were provided with detailed information about the purpose, procedures, risks, and benefits of the research, ensuring their decision to participate was fully informed. Written consent was obtained from all participants. They were assured of their voluntary participation and their right to withdraw from the study at any time without penalty. All personal data were anonymized to maintain confidentiality. Standardized protocols for data collection and analysis were employed to ensure objectivity and minimize potential biases in the interpretation of the results

## Data Availability

Zenodo: Relationship between corporate culture, service quality, and customer satisfaction of food and beverage franchise enterprises; https://doi.org/10.5281/zenodo.14751644 ( Chau Thi Le et al., 2025). This project contains the following data:
• Figures F&B Corporate culture.xlsx Zenodo: Relationship between corporate culture, service quality, and customer satisfaction of food and beverage franchise enterprises, https://doi.org/10.5281/zenodo.14751644 ( Chau Thi Le et al., 2025). This project contains the following data:
• Questionaire-Corporate culture.docx Data are available under the terms of the Creative Commons Attribution 4.0 International license (CC-BY 4.0).
