# Supplementary material for: Relationship between corporate culture, service quality, and customer satisfaction of food and beverage franchise enterprises
Source: F1000Res. 2025 Mar 5;14:267. [Version 1] doi: 10.12688/f1000research.154431.1 (PMC12120415; doi:10.12688/f1000research.154431.1)
Supplement: Peer review history

# Reviewer report: Reviewer response for version 1

**DOI:** 10.5256/f1000research.169462.r381452

**Published:** 2025-05-28

**Authors:** Dr. Fathorrahman SE, MM

**Affiliations:** 1 Human Resource Management, Institut Teknologi dan Bisnis Asia Malang, East Java, Indonesia

1. This background does not adequately explain the role of service quality in influencing customer satisfaction.

 It is necessary to explain why the researchers examined the role of service quality as a mediating variable in this study.

 2. Many of the literature references are quite old. It also needs to be updated with the latest literature.

 3. There is no hypothesis regarding service quality as a mediating variable in the influence of corporate culture on customer satisfaction. 

 4. Researchers need to explain how they selected respondents to be included as research samples.

 5. The SEM output analysis results show that the quality of service acts as a mediating variable. ?

Is the work clearly and accurately presented and does it cite the current literature?

Partly

If applicable, is the statistical analysis and its interpretation appropriate?

Yes

Are all the source data underlying the results available to ensure full reproducibility?

Yes

Is the study design appropriate and is the work technically sound?

Yes

Are the conclusions drawn adequately supported by the results?

Yes

Are sufficient details of methods and analysis provided to allow replication by others?

Yes

Reviewer Expertise:

human resource management and marketing management

I confirm that I have read this submission and believe that I have an appropriate level of expertise to confirm that it is of an acceptable scientific standard, however I have significant reservations, as outlined above.

# Reviewer report: Reviewer response for version 1

**DOI:** 10.5256/f1000research.169462.r381455

**Published:** 2025-05-28

**Authors:** Josef Herget

**Affiliations:** 1 Excellence Institute—Research & Solutions, Larnaca, Cyprus

This article addresses a highly relevant topic—the relationship between corporate culture, service quality, and customer satisfaction—within the underexplored context of Vietnam’s food and beverage franchise sector. The study is well-structured and methodologically sound, employing Structural Equation Modeling (SEM) with appropriate reliability and validity checks. The empirical setting adds value to the literature, particularly in showcasing how culture influences service quality in franchise operations in emerging markets.

 The study's strengths include its practical relevance, solid analytical execution, and contextual novelty. The authors provide clear implications for practitioners, notably regarding the role of symbolic culture (e.g., uniforms, logos) and customer-centered service processes. The use of Schein’s three-layer model is appropriate but could be more deeply explored. In this survey it is limited merely to artifacts, so this research cannot claim to provide knowledge on the relationship between corporate culture and service quality (and customer satisfaction). The values, beliefs, underlying assumptions are not part of the questionnaire. More to this, this part should be (additonally) analyzed by the staff of the respective businesses and not by the consumers. Summarizing: the mechanisms through which corporate culture affects service quality and customer satisfaction are not sufficiently theorized. For instance, psychological safety, empowerment, or organizational learning could be used to explain how culture translates into front-line behaviors. The sampling strategy is not described in detail as well. Was convenience or random sampling used? Were the respondents employees, managers, or customers?

 To enhance the paper’s academic contribution, I recommend deepening the theoretical framework and clarifying the mechanisms linking corporate culture to customer satisfaction. The discussion section would benefit also from a stronger comparison with international research and a more critical interpretation of findings. Additionally, minor issues—such as typographical errors, inconsistent terminology (e.g., “qualitity”) or not referenced explanations like "Duong Thi Lieu has also employed this model in her studies" (which studies?), and lack of detail on the sampling strategy—should be corrected for clarity and rigor.

 I also suggest the authors include more information on the specific items used to measure key constructs (e.g., corporate culture, service quality) to support replication and transparency. Finally, recommendations for future research should be explicitly addressed in the conclusion.

 Recommendations for Improvement

1. **Deepen the theoretical framework**: Draw more clearly on established models of organizational culture and service quality and explore alternative conceptual pathways (e.g., cultural fit, trust, emotional labor). The paper would benefit from a more nuanced engagement with existing theories of organizational culture (e.g., Hofstede’s cultural dimensions, Cameron & Quinn’s Competing Values Framework) and service quality (e.g., Grönroos, customer journey mapping). The authors rely heavily on Schein’s three-level model but do not connect it robustly to mechanisms that influence frontline service behavior or customer perception. The mediating role of service quality is supported by empirical results but would benefit from more theoretical elaboration in the literature review. 
2. **Clarify the sample and data collection procedures**: Explain who the respondents are, how they were selected, and how generalizable the results are. What are the differences in their categories (male/female, first time customers vs. frequent buyers ....)
3. **Address potential biases:** Discuss how common method variance was mitigated (e.g., procedural remedies, Harman’s test).
4. **Include measurement items**: Provide an appendix or table that details the questionnaire items used for each construct (do not hide it in an external link).
5. **Enhance critical discussion**: Reflect on findings in the light of global literature. What is unique about the Vietnamese context? What lessons travel beyond it?
6. **Reframe the contribution**: Is this study a validation, an extension, or a contradiction of established theory? Clarify the position.

**Overall, I recommend minor revision.** This paper offers a valuable empirical contribution and should be indexed after improvements in theoretical articulation and language accuracy.

Is the work clearly and accurately presented and does it cite the current literature?

Partly

If applicable, is the statistical analysis and its interpretation appropriate?

Partly

Are all the source data underlying the results available to ensure full reproducibility?

Yes

Is the study design appropriate and is the work technically sound?

Partly

Are the conclusions drawn adequately supported by the results?

Partly

Are sufficient details of methods and analysis provided to allow replication by others?

Partly

Reviewer Expertise:

Management Science, Corporate Culture, Information Science

I confirm that I have read this submission and believe that I have an appropriate level of expertise to confirm that it is of an acceptable scientific standard, however I have significant reservations, as outlined above.

# Reviewer report: Reviewer response for version 1

**DOI:** 10.5256/f1000research.169462.r376449

**Published:** 2025-05-24

**Authors:** Tin Agustina Karnawati

**Affiliations:** 1 Institut Teknologi Dan Bisnis Asia, Malang, Indonesia

Relationship between corporate culture, service quality and customer satisfaction of food and beverage franchise enterprises

 Duyen Chau Thi Le, Liem Nguyen Thanh, Linh Huynh Giao

 I think this article is excellent for enriching the literature review on customer satisfaction. It discusses the relationship between corporate culture and service quality on customer satisfaction in food and beverage franchise enterprises in the Mekong Delta region of Vietnam. The topic of customer satisfaction remains interesting to study, even though there has been a lot of research on it. However, it is hoped that this research can make an important contribution to consumer studies, especially on the object of food and beverage companies that are constantly faced with competition and demands for innovation in their services.

 This paper is already very comprehensive in its presentation of the results, which are strengthened by very adequate statistical processing. The following are more specific comments based on the sections:

**Abstract**

 The background section needs clarification on the issues supporting the necessity of understanding the relationship between corporate culture, service quality, and customer satisfaction. The methodology adequately describes the research design; however, it would be better to add who the research subjects were. The results clearly demonstrate the influence between variables, but it is advisable to be consistent with the term "corporate culture" rather than "organizational culture," even though their meanings are almost the same. In the Conclusion section, it is suggested to add the contribution or what suggestions there are for future research.

**Background**

 The background presentation has reviewed the overview of the franchise sector and comparisons with other international brands. However, it needs a slightly more detailed discussion of the key issues that more directly relate to what aspects of satisfaction are being examined. It would be more in-depth and add to the topic's appeal if a discussion of the research gap were included, highlighting what problems remain unanswered in previous research or are still being debated, thus indicating the continued need for research on the variables in this study.

**Theoretical Background and Research Model**

 This section is already very good at explaining the relationship between the research variables, also revealing the explanation within the reference to the service quality model with SERVQUAL. The explanation of customer satisfaction has also been well-reviewed, but it needs to add how corporate culture plays a role here. Its relationship hasn't been elaborated, which would serve as an initial support for the hypotheses and connect with Figure 1 of the research model. On page 4, the spelling of the word "quality" in hypotheses H1 and H2 needs to be corrected by the author.

**Methodology**

 The methodology section is very comprehensive in its explanation. However, the writing of sentences in the "ethical considerations" subsection on page 5 needs correction. Several phrases like "accessvalidity," "minimizeThus," "torecommendsusedAdditinally," "5every," "meaningideally," and "ofsuggest" need to be revised for correct spelling. This section should also be double-checked against page 12.

**Results**

 The explanation of the analysis results is already sufficient, supported by data processing tables and the explanation of hypothesis acceptance. However, it needs an additional discussion comparing the findings with relevant empirical findings or previous research.

**Conclusion**

 It has been adequately presented; however, it would be better if recommendations for future research were added.

Is the work clearly and accurately presented and does it cite the current literature?

Partly

If applicable, is the statistical analysis and its interpretation appropriate?

Yes

Are all the source data underlying the results available to ensure full reproducibility?

Yes

Is the study design appropriate and is the work technically sound?

Yes

Are the conclusions drawn adequately supported by the results?

Yes

Are sufficient details of methods and analysis provided to allow replication by others?

Yes

Reviewer Expertise:

HR Management and Marketing

I confirm that I have read this submission and believe that I have an appropriate level of expertise to confirm that it is of an acceptable scientific standard.
